# Supplementary material for: AgentMD: Empowering language agents for risk prediction with large-scale clinical tool learning
Source: Nat Commun. 2025 Oct 23;16:9377. doi: 10.1038/s41467-025-64430-x (PMC12549800; doi:10.1038/s41467-025-64430-x)
Supplement: Supplementary file 1 — Supplementary Information [file 41467_2025_64430_MOESM1_ESM.pdf]

Supplementary Materials for “AgentMD: Empowering Language Agents for Risk Prediction with Large-Scale Clinical Tool Learning”

RiskCalcs – AgentMD Tool Creation

In Supplementary Figure 1, we show a detailed pipeline for building the RiskCalcs tool collection. The first step (Supplementary Figure 1a) is to filter over 37 million PubMed abstracts for potential risk calculators with a Boolean query: "patient" AND ("risk" OR "mortality") AND ("score" OR "point" OR "rule" OR "calculator"). About 340 thousand articles passed the initial screen. We then used GPT-3.5 to further screen articles that describe a new risk score or risk calculator with Prompt #1 (Supplementary Table 1), which leads to about 33 thousand articles for tool creation (Supplementary Figure 1b).

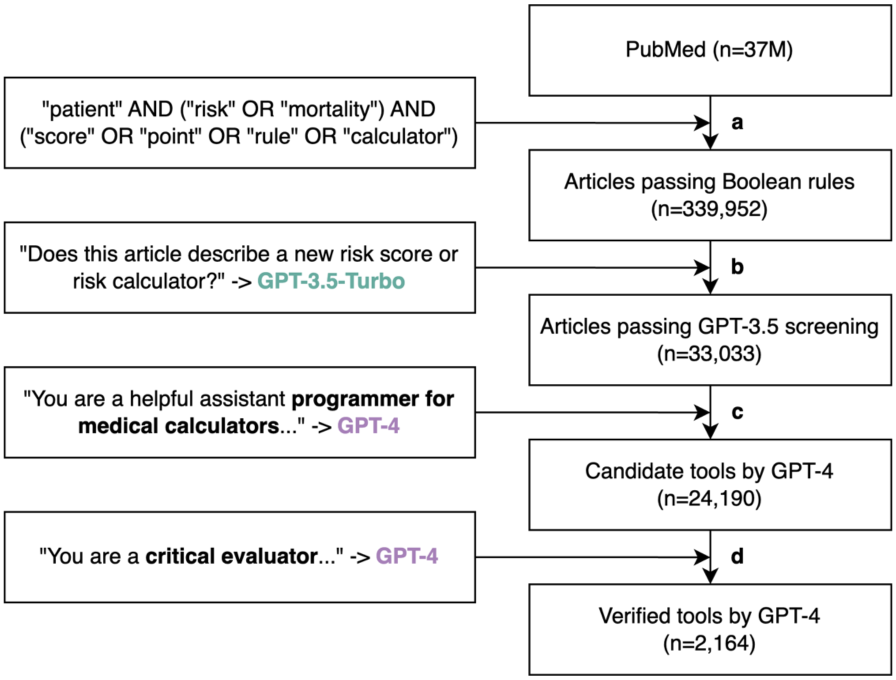

Supplementary Figure 1. The pipeline of building RiskCalcs. a, All PubMed abstracts are first filtered by a Boolean query for potential risk calculators. b, GPT-3.5-Turbo further screened the articles for ones that describe a new risk score or calculator. c, GPT-4 drafts candidate calculators for the articles that have passed GPT-3.5 screening. d, The drafted calculators are then verified by GPT-4, and 2,164 calculators have passed the verification and are included in RiskCalcs.

We used GPT-4 to generate the RiskCalcs tools in two steps: the first step (Supplementary Figure 1c) is to create the candidate tools given the PubMed article with Prompt #2 (Supplementary Table 2) and one example is shown in Supplementary Figure 2a; the second step (Supplementary Figure 1d) is to verify the generated calculators with a list of quality-checking questions (using Prompt #3 as shown in Supplementary Table 3), and only calculators that passed all verification questions are included in the RiskCalcs collection. Overall, GPT-4 has generated 24,190 tools, where 2,164 tools have passed the verification and have been included in the RiskCalcs collection.

Supplementary Table 1. Prompt #1 for screening PubMed articles for potential calculators.

User

Here is a PubMed article:

{PubMed article title}

{PubMed article abstract}

Does this article describe a new risk score or risk calculator? In healthcare, a risk score quantitatively estimates the probability of a clinical event or outcome, such as disease development or progression, within a specified period. These scores are derived from algorithms using variables like patient demographics, clinical history, laboratory results, and other relevant health indicators. They aid clinicians in decision-making, allowing for personalized patient care and resource allocation. Simply answer with “yes” or “no”:

GPT-3.5-Turbo

{Output}

Supplementary Table 2. Prompt #2 for RiskCalcs Tool Creation.

System

You are a helpful assistant programmer for medical calculators. Your task is to read a PubMed article about a medical calculator, and if applicable, write a two-step calculator: (1) calculate a risk score based on multiple criteria; (2) interpret different ranges of the computed risk score into probabilities of risks.

User

Here is a PubMed article:

{PubMed article title}

{PubMed article abstract}

Does the article describe a simple two-step risk calculator, where the first step is to compute a risk score, and the second step is to interpret different risk scores? If no, please directly and only output "NO". Otherwise, please standardize the calculator into:

#Title \n The name of the calculator(s).

##Purpose \n Describe when this calculator should be used.

##Specialty \n should be a list of calculator types, one or more of {specialty list}, separated by ",".

##Eligibility \n Describe what patients are eligible.

##Size \n The exact number of patients used to derive this calculator. Only put a number here without any other texts.

##Computation \n Detailed instructions of how to use the calculator, including Python functions with clear docstring documentation. Please be self-contained and detailed. For example, if the

computation involves multiple items, please clearly list each item. If one item has multiple possible values (e.g., 0-2), you also need to clearly define what each value means.

##Interpretation \n Should be a list, where each item describes the interpretation (actual risk) for a value or a range of the computed risk score.

##Utility \n Evaluation results of the clinical utility of the risk score, such as AUC, F-score, PPV.

##Example \n Generate a sample patient note and a detailed demonstration of using the calculator and interpret the results. Think step-by-step here.

Please be as detailed as possible.

GPT-4

{Output}

Supplementary Table 3. Prompt #3 for verifying the curated calculator tools.

System

You are a critical evaluator for a calculator that's supposed to describe a PubMed article. The calculator might contain errors. Always respond in a JSON dict formatted as Dict{“reasoning”: Str(critical_reasoning), “answer”: Str(yes/no)}.

User

Here is the original PubMed article:

{PubMed article title}

{PubMed article abstract}

Here is the calculator that's supposed to describe the article above:

{Generated calculator}

Are the parameters clearly defined in the #Computation? If a parameter can have different scores, the definitions for each score must be provided.

Are the parameters defined exactly the same in the article and the calculator?

Is the #Computation logic in the calculator fully based on the original article without any assumptions? Answer no if the article does not provide clear computing logics or weights.

Is the #Interpretation of the calculator fully based on the original article without any assumptions? Score ranges and corresponding risks should be exactly the same between the calculator and the article.

Is the #Interpretation of the calculator useful? A useful calculator should contain quantitative risk rates or qualitative risk groups for different score ranges.

Is the calculator free from any bug or other issue?

GPT-4

{Output}

Characteristics of the RiskCalcs Toolkit

Each calculator is classified by GPT-4 into one or more of the ten human organ systems in Medical Subject Headings (MeSH): Musculoskeletal (A02), Digestive (A03), Respiratory (A04), Urogenital (A05), Endocrine (A06), Cardiovascular (A07), Nervous (A08), Stomatognathic (A14), Hemic and

Immune (A15), and Integumentary (A17) Systems. Supplementary Figure 2b shows the total number, PubMed article citation and population distributions of calculators in different systems. Overall, the cardiovascular system has the most calculators (811), and the stomatognathic system has the least (36). Although different systems differ in the number of calculators available, the distributions of total article citations and cohort sizes remain similar.

Supplementary Figure 2c shows the t-SNE visualizations [1] of the semantic representations of these calculators derived from MedCPT [2]. Each point represents a calculator and is color-coded by the organ system shown in Supplementary Figure 2b. The clustering results show that calculators of the same organ systems generally have similar semantic representations, suggesting the effectiveness of our topic classification system and the diversity of the clinical calculators in RiskCalcs.

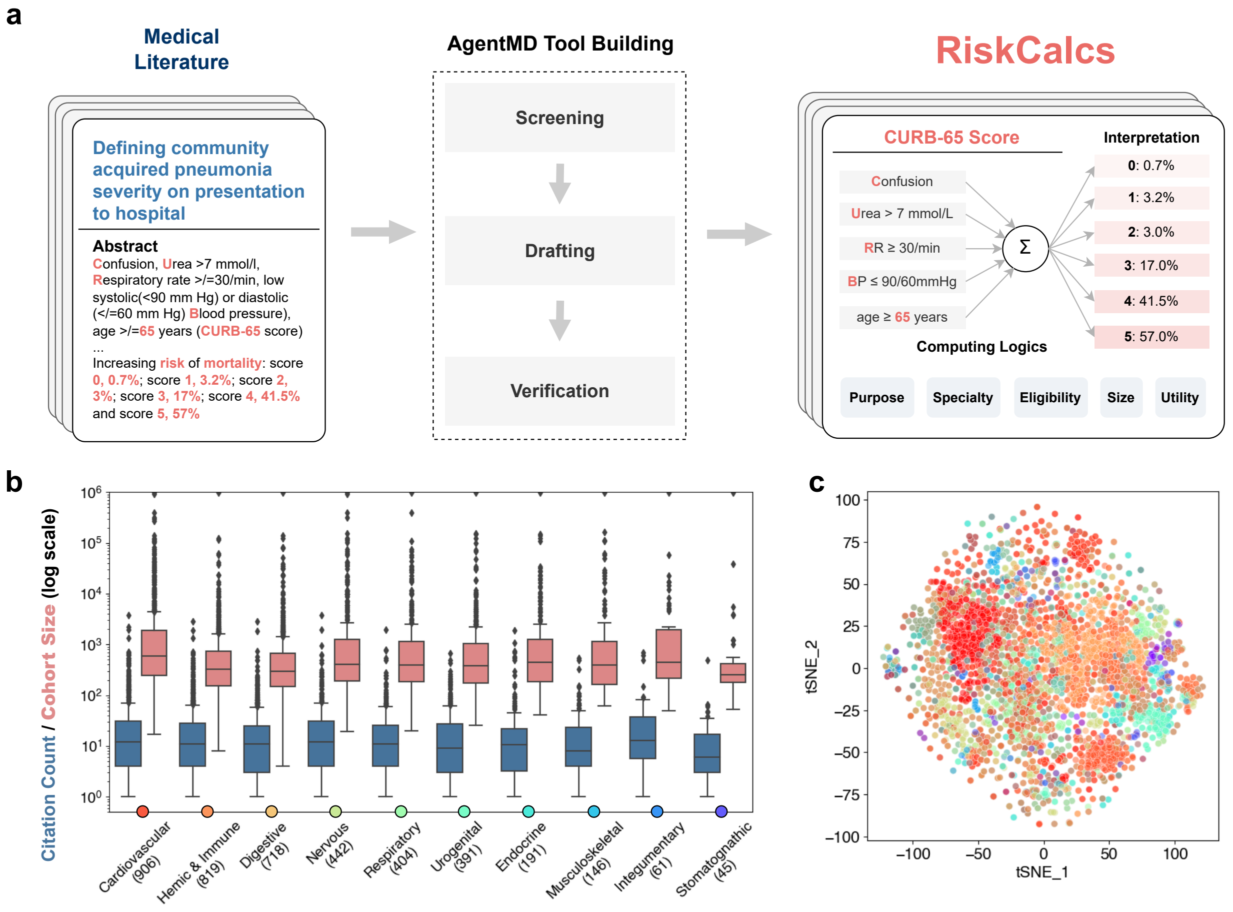

Supplementary Figure 2. Overview of the RiskCalcs characteristics. **a**, The process of building the RiskCalcs tool collection. AgentMD screens PubMed abstracts, drafts initial versions of the tools,

and further verifies them. Only the verified calculators are included in RiskCalcs. **b**, The distribution of article citation count and calculator cohort size within the calculators of different organ systems. Lower quartile, median, and upper quartile are shown in the box plots. Outliers beyond 1.5 interquartile range are also displayed. **c**, t-SNE visualizations of the semantic representations of tools in RiskCalcs.

RiskQA Evaluation

RiskQA contains 350 United States Medical Licensing Examination (USMLE)-style [3] multi-choice question answering instances, where the questions are descriptions of patients and the answer options are different quantitative outcome measures (e.g., mortality). Under the standard RiskQA setting, AgentMD performs tool selection (with MedCPT and Prompt #4, shown in Supplementary Table 4), tool computation (with Prompt #5, shown in Supplementary Table 5), and finally generates the answer. For the Chain-of-thought (CoT) [4] baseline, we appended "Let's think step-by-step" after the whole question as the prompt to LLMs.

Supplementary Table 4. Prompt #4 for AgentMD tool selection in RiskQA.

**User**<br><br>Please choose the most appropriate tool from the listed ones to solve the question below:<br><br>{RiskQA question}<br><br>10 × [<br>Tool ID: {Tool PMID}; Title: {Tool #Title}; Purpose: {Tool ##Purpose}<br>]<br><br>Please copy the most appropriate tool:<br><br>**GPT-4**<br><br>{Output}

Supplementary Table 5. Prompt #5 for AgentMD tool computation in RiskQA.

System

You are a helpful assistant. Your task is to apply a medical calculator to solve an USMLE-style question. You can write Python scripts and the user will execute them for you. The Python function in the calculator has already been in the environment, which you can re-use or revise if there is bug. Your responses will be used for research purposes only. Please start with "Answer: " to choose the answer if you have finished the task. Please choose the closest answer if there is no exact match.

User

Here is the calculator:

{RiskCalcs calculator}

Here is the USMLE question:

{RiskQA question}

Please apply this calculator to the patient. Please write Python scripts and `print()` the results to help the computation. I will provide the stdout to you.

N × [

GPT-4

{Output}

Python Interpreter

{Output}

]

GPT-4

{Output}

Emergency Department Note Evaluation

With a pre-selected list of emergency care calculators, AgentMD bypasses the retrieval step and directly selects the eligible ones from the provided list using Prompt #6 (Supplementary Table 6) as the first step. Then, AgentMD will apply the selected calculators to the given patient by interacting with a Python Interpreter, using Prompt #7 (Supplementary Table 7). Prompt #7 is similar to Prompt #5 with slight modifications to handle the missing values in real-life clinical notes. The final tool computation results will be further processed by Prompt #8 (Supplementary Table 8) to get a numeric score for each patient-calculator pair that will be used for ranking and choosing the most risky patients for the manual evaluations.

Supplementary Table 6. Prompt #6 for AgentMD tool selection for the ED evaluation.

System

You are a helpful assistant and your task is to select the calculators that a given patient is eligible for. Here are the candidate calculators:

{Calculators}

Please first explain what calculators a given patient is eligible for, and then output the list of calculator IDs. Please output a JSON dict formatted as Dict{"explanation": Str(explanation), "calculators": List[Int(ID)]}. Please be strict.

User

Here is the patient note:

{Note}

Output in JSON:

GPT-4

{Output}

Supplementary Table 7. Prompt #7 for AgentMD tool computation for the ED evaluation.

System

You are a helpful assistant. Your task is to apply a medical calculator to a patient and interpret the result. You can write Python scripts and the user will execute them for you. The Python function in the calculator has already been in the environment, which you can re-use or revise if there is bug. Your responses will be used for research purposes only. Please start with \"Summary: \" to summarize the messages in one paragraph if you have finished the task. Please make sure to include the raw results of the calculator in the summary.

User

Here is the calculator:

{Emergency care calculator}

Here is the patient information:

{Emergency department provider note}

Please apply this calculator to the patient. If there are missing values, please make a range estimation based on best and worst case scenarios inferred from the calculator computing logics. Please write Python scripts and print the results to help the computation. I will provide the stdout to you.

N × [

GPT-4

{Output}

Python Interpreter

{Output}

]

GPT-4

{Output}

Supplementary Table 8. Prompt #8 for AgentMD tool scoring for the ED evaluation.

System

You are a helpful medical assistant, and your task is to give an overall score (0-100) given a summary of medical calculation. Higher scores denote more urgent and severe conditions that

require immediate attention. If the calculation result contains a wide range, give a low score due to its uncertainty.

User

Here is the summary:

{AgentMD tool computation results}

Output only the overall score (0-100):

MIMIC-III Evaluation

To preprocess the MIMIC-III dataset [5], we used the scripts provided by a previous study to get its test set admission notes [6]. We applied AgentMD to the test split of 9,822 patients. Experiments on the MIMIC-III dataset uses a slightly modified tool selection because various risks should be considered for each patient. As such, AgentMD first uses LLMs to generate a list of potential risk descriptions for the given patient admission note with Prompt #9 (Supplementary Table 9). Then, AgentMD applies tool selection to each generated risk using Prompt #10 (Supplementary Table 10), where the patient representation is computed by encoding the textual description of the risks with MedCPT. Finally, AgentMD uses Prompt #11 (Supplementary Table 11) to conduct the tool computation given the patient note and a selected tool. After AgentMD has computed the results of a given patient-calculator pair, it will further use Prompt #12 (Supplementary Table 12) to evaluate the specificity, urgency, severity, and absence in the note of the risk results for the patient. These scores are used to draw the calculator result distributions in Figure 5e.

Supplementary Table 9. Prompt #9 for initial risk generation for MIMIC-III patients.

System

You are a helpful assistant doctor. Your task is to generate a list of risks for the given patient. Your response is for research purpose only and will not be used in clinical practice.

User

Here is the patient admission note:

{Patient admission note}

Please generate a list of 5 potential clinical risks that are significant, urgent, and specific to the patient. Output a json list where each element is a self-contained short risk string that contains both the risk event and the underlying condition, e.g. "X due to Y". Please be concise, and each risk should only contain several words.

GPT-4

{Output}

Supplementary Table 10. Prompt #10 for AgentMD tool selection for MIMIC-III patients.

System

You are a critical evaluator. Your task is to judge whether the given patient belongs to the eligible population of the given medical calculator. Your response will be used for research purposes only.

User

Here is the patient admission note:

{Patient admission note}

Here is the calculator:

{RiskCalcs calculator}

Please think step-by-step and then judge whether (1) whether the patient is eligible to use the calculator; (2) whether all parameters for the calculators are missing in the patient note. Output a json dict formatted as Dict{"step_by_step_reasoning": Str(...), "patient_eligible": Str(yes|no), "missing_all_parameters": Str(yes|no)}.

GPT-4

{Output}

Supplementary Table 11. Prompt #11 for AgentMD tool computation for MIMIC-III patients.

System

You are a helpful assistant. Your task is to apply a medical calculator to an imaginary patient and interpret the result. You can write Python scripts and the user will execute them for you. The Python function in the calculator has already been in the environment, which you can re-use or revise if there is bug. Your responses will be used for research purposes only. Please start with "Summary: " to summarize the messages in one paragraph if you have finished the task. Please make sure to include the raw results of the calculator in the summary.

User

Here is the calculator:

{RiskCalcs calculator}

Here is the patient information:

{Patient admission note}

Please apply this calculator to the patient. If there are missing values, please make a range estimation based on best and worst case scenarios inferred from the calculator computing logics. Please write Python scripts and print the results to help the computation. I will provide the stdout to you.

N × [

GPT-4

{Output}

Python Interpreter

{Output}

]

GPT-4

{Output}

Supplementary Table 12. Prompt #12 for AgentMD patient ranking for MIMIC-III patients.

System

You are a helpful assistant for a hospital warning system. Your task is to determine the specificity, urgency, severity, and absence of a risk calculator result applied to a patient. Your response will be used for research purposes only.

User

Here is the patient note:

{Patient admission note}

Here is the calculator result:

{AgentMD calculation result}

Specificity (0-100) denotes the confidence of the calculator result. Specificity is low if there are missing values and the range is wide between the risk scores of the best-case and worst-case scenarios. Specificity is high if there is no range estimation (best and worse case scenarios) and the risk calculator result contains only one specific score (not a range)

Urgency (0-100) denotes whether the risk considered by the calculator is acute or chronic. Urgency is high if there is immediate danger worth medical attention. On the other hand, urgency is low if the risk is about 1-year or 5-year.

Severity (0-100) denotes the extent of the calculated risk. Severity is high if the predicted risk probability is close to 100%. Severity is low if the predicted risk probability is close to 0%.

Absence (0-100) denotes whether the calculated risk is missing in the original patient note. Absence is 100 if the calculator result (the risk, not the calculator name) is not considered or reflected in the patient note. Absence is 0 if the calculator result has already happened or been considered in the patient note.

Please be critical and only output a json dict formatted as: {"rationale": Str(detailed_explanations_for_all_scores), "specificity": float(0-100), "urgency": float(0-100), "severity": float(0-100), "absence": float(0-100)}.

GPT-4

{Output}

Analysis of Structured Parameter Mapping

We analyzed the 16 clinical calculators used in our emergency department evaluation and found that the majority (11 out of 16, 68.75%) require at least one unstructured input parameter that lacks representation in standard terminologies such as Logical Observation Identifiers Names and Codes (LOINC). The results are shown in Supplementary Table 13. For example, the HEART score includes the criterion “positive family history (parent or sibling with CVD before age 65)”, which is not easily mapped to an ontology term.

Supplementary Table 13. List of parameters across 16 clinical calculators. Parameters were determined as structured or unstructured using LOINC keyword search (https://loinc.org/search).

**Calculator**	**Parameters**	**Number of parameters**	**Parameters that CANNOT be mapped to standard codes**
Pulmonary Embolism Rule-out Criteria	age, pulse, oxygen saturation, unilateral leg swelling, hemoptysis, recent trauma or surgery, prior PE or DVT, hormone use	8	4
X-ray Need	Pain location, bone tenderness tibia, bone tenderness fibula, inability to bear weight	4	3

Canadian C-Spine Rule	age, GCS score, trauma mechanism, paraesthesia extremities, rear end collision, sitting position, ambulatory, delayed neck pain, no midline tenderness, neck rotation	10	7
Fracture Rule	age, tenderness at fibula head, isolated patella tenderness, inability to flex 90 degrees, inability to bear weight	5	4
DVT Wells' Score	active cancer, calf swelling, unilateral superficial veins, unilateral pitting edema, previous DVT, entire leg swelling, local tenderness, recent immobilization or surgery, alternative diagnosis	9	8
PE Diagnosis Score	DVT symptoms, alternative diagnosis likelihood, heart rate, immobilization or surgery, previous DVT or PE, hemoptysis, malignancy with treatment	7	5
Centor Score	temperature, cough, tonsillar swelling, anterior cervical adenopathy, age	5	2
Low Risk Score	age, glasgow score, normal mental status, scalp hematoma, loss of consciousness, injury mechanism, skull fracture, acting normally, vomiting, signs of skull fracture, severe headache	11	6
CURB-65 Mortality Score	confusion, urea, respiratory rate, systolic pressure, diastolic pressure, age	6	0

HEART Score	history, ECG abnormality, age, risk factors, troponin level	5	2
CHADS2 Index	had congestive heart failure, had hypertension, age, has diabetes, had stroke or TIA	5	0
GCS	eye score, verbal score, motor score	3	0
CT Head Rule	gcs score 15 in 2h fail, age, suspected open skull fracture, sign of basal skull fracture, vomiting episodes, amnesia before impact, dangerous mechanism of injury	7	4
qSOFA Score	systolic BP, respiratory rate, altered mentation	3	0
Glasgow Blatchford Bleeding Score	hemoglobin, BUN, systolic BP, sex, heart rate, melena present, recent syncope, hepatic disease history, cardiac failure	9	4
SIRS criteria	temperature, heart rate, respiratory rate, PaCO2, white blood cell count	5	0

Analysis of the Impacts of Structured Data

We have also conducted an additional analysis of the effects of structured data using the MIMIC-III multi-task learning package [7]. Specifically, we appended the first appearance value of the processed structured data in the first 48 hours to the end of the admission note. Such structured data covers source from labs tests, vital signs, bedside monitors, and includes: capillary refill rate, diastolic blood pressure, fraction of the inspired oxygen, Glascow coma scale (eye opening), Glascow coma scale (motor response), Glascow coma scale (verbal response), Glascow coma scale (total), glucose, heart rate, height, mean blood pressure, oxygen saturation, respiratory rate, systolic blood pressure, temperature, weight, and pH. We compare the differences of AgentMD's predictions for the

same patient-calculator pairs between using only the admission note and using the admission note with the appended structured data. Our results show that: First, structured data significantly improves the specificity of AgentMD's predictions (from 62.2% to 69.6%, paired t-test p=2.2e-173), which shows that some missing input parameters in the admission notes can be retrieved from the structured data. Second, the majority of tools (54.6%) show better or the same AUC performance for mortality prediction after adding the structured data, which further indicates the potential utility of the structured data.

Supplementary References

1. Van der Maaten, L. & Hinton, G. Visualizing data using t-SNE. *Journal of machine learning research* **9**(2008).
2. Jin, Q., *et al.* MedCPT: Contrastive Pre-trained Transformers with large-scale PubMed search logs for zero-shot biomedical information retrieval. *Bioinformatics* **39**, btad651 (2023).
3. Jin, D., *et al.* What disease does this patient have? a large-scale open domain question answering dataset from medical exams. *Applied Sciences* **11**, 6421 (2021).
4. Wei, J., *et al.* Chain of thought prompting elicits reasoning in large language models. *arXiv preprint arXiv:2201.11903* (2022).
5. Johnson, A.E., *et al.* MIMIC-III, a freely accessible critical care database. *Sci Data* **3**, 160035 (2016).
6. Van Aken, B., *et al.* Clinical Outcome Prediction from Admission Notes using Self-Supervised Knowledge Integration. in *Proceedings of the 16th Conference of the European Chapter of the Association for Computational Linguistics: Main Volume* 881-893 (2021).
7. Harutyunyan, H., Khachatrian, H., Kale, D.C., Ver Steeg, G. & Galstyan, A. Multitask learning and benchmarking with clinical time series data. *Scientific data* **6**, 96 (2019).
